# Supplementary material for: Current protected areas provide limited benefits for European river biodiversity
Source: Nat Commun. 2025 Dec 17;16:11146. doi: 10.1038/s41467-025-67125-5 (PMC12711900; doi:10.1038/s41467-025-67125-5)
Supplement: Supplementary file 1 — Supplementary Information [file 41467_2025_67125_MOESM1_ESM.pdf]

Supplementary Table 1 River invertebrate time series information by country. Includes the number of sites, the earliest and latest sampling years, the mean number of sampling years and total time series duration across sites, and the invertebrate sampling methods and abundance units.

Country	Number of sites	Start year	End year	Sampling years	Total duration	Sampling method(s)	Abundance units
Belgium	60	1989	2021	10.6	23.3	Multi-habitat kick net (0.3–0.5mm mesh) or colonization of artificial substrates	Ind. per sample
Czechia	109	2000	2021	8.8	14.8	Multi-habitat kick net (0.5mm mesh)	Ind. per sample
Denmark	233	1992	2018	20.5	22.6	Multi-habitat kick net (0.5mm mesh)	Ind. per 0.6 m^2
Finland	55	1998	2021	10.2	15.1	Multi-habitat kick net (0.5mm mesh)	Ind. per sample
France	463	1992	2022	12.3	17.8	Multi-habitat Surber sampler (0.5mm mesh)	Ind. per 0.4 m^2
Hungary	64	2005	2019	11.0	12.1	Multi-habitat kick net (0.5mm mesh)	Ind. per m^2
Lithuania	31	2010	2020	8.2	11.0	Multi-habitat kick net (0.5mm mesh)	Ind. per sample
Spain	179	1986	2021	19.8	21.9	Multi-habitat kick net (0.5-mm mesh)	Ind. per 5 subsamples
Sweden	82	1995	2019	14.3	14.7	Multi-habitat kick net (0.5-mm mesh)	Ind. per m^2

UK	478	1991	2019	12.4	23.1	Multi-habitat kick net (1-mm mesh)	Ind. per sample

Supplementary Table 2 Results from all linear mixed models testing differences in rates of abundance, richness, and EQR change between protected and unprotected sites, and sites that gained and did not gain upstream PAs. Note the degrees of freedom (i.e., number of parameters that differ between models with and without the categorical PA term) were always 1, and *P*-values are provided after false discovery rate correction.

**Metric**	**Model**	**Upstream scale**	**L**	***P***
Abundance	Protected and unprotected	1 km	0.23	0.84
		10 km	0.15	0.84
		100 km	2.40	0.32
		Full	1.41	0.47
	Gain and no gain	1 km	0.12	0.84
		10 km	0.02	0.89
		100 km	4.80	0.19
		Full	3.94	0.19
Richness	Protected and unprotected	1 km	4.02	0.087
		10 km	5.96	0.059
		100 km	3.70	0.087
		Full	3.00	0.11
	Gain and no gain	1 km	2.74	0.11
		10 km	1.60	0.20
		100 km	5.45	0.059
		Full	5.25	0.059
EQRs	Protected and unprotected	1 km	23.37	0.000011*
		10 km	5.96	0.039*
		100 km	0.35	0.88
		Full	0.17	0.88
	Gain and no gain	1 km	7.80	0.021*
		10 km	1.60	0.88
		100 km	0.0012	0.97
		Full	0.084	0.88

Supplementary Table 3 Wald-type test results from all models of richness and ecological quality (EQR), including effective degrees of freedom (edf), of the significance ($P < 0.05$) of smoothed interaction terms between protected areas (PA) and river size (RS; log-transformed km^2), and between protected areas and initial river quality (EQR_i). *P*-values are provided after false discovery rate correction.

**Metric**	**Model**	**Upstream area**	**Interaction**	**edf**	**F**	***P***
Richness	PA cover	1 km	PA:RS	1.00	1.00	0.86
			PA:EQR_i	7.23	4.05	0.0024*
		10 km	PA:RS	1.00	0.003	0.97
			PA:EQR_i	3.20	6.26	0.0024*
		100 km	PA:RS	1.56	0.65	0.76
			PA:EQR_i	1.79	5.58	0.020*
		Full	PA:RS	1.63	0.50	0.69
			PA:EQR_i	1.00	7.04	0.032*
	PA gain	1 km	PA:RS	2.25	0.86	0.51
			PA:EQR_i	2.14	1.25	0.51
		10 km	PA:RS	1.64	0.46	0.75
			PA:EQR_i	3.29	2.14	0.18
		100 km	PA:RS	1.57	0.49	0.77
			PA:EQR_i	2.59	3.35	0.077
		Full	PA:RS	1.00	0.48	0.71
			PA:EQR_i	1.00	6.31	0.039*
EQR	PA cover	1 km	PA:RS	1.00	3.26	0.13
			PA:EQR_i	1.00	5.47	0.045*
		10 km	PA:RS	2.01	2.36	0.14
			PA:EQR_i	11.58	3.92	0.000029*
		100 km	PA:RS	1.80	1.22	0.54
			PA:EQR_i	3.64	11.70	0.00*
		Full	PA:RS	1.97	0.80	0.58
			PA:EQR_i	3.57	10.63	0.000017*
	PA gain	1 km	PA:RS	1.00	0.45	0.58

	PA:EQR_i	1.00	0.33	0.60
10 km	PA:RS	1.00	0.0010	0.98
	PA:EQR_i	4.47	10.31	0.00*
100 km	PA:RS	2.40	1.99	0.21
	PA:EQR_i	12.02	7.57	0.00*
Full	PA:RS	4.18	2.46	0.084
	PA:EQR_i	12.33	7.96	0.00*

Supplementary Table 4 Methods used to produce the Ecological Quality Ratios for Belgium, Czechia, Denmark, Finland, France, Hungary, Lithuania, Spain, Sweden, and the UK.

**Country**	**Method**	**Reference(s)**
Belgium	Multimetric Macroinvertebrate Index Flanders	1
Czechia	Multimetric index using river type-specific metrics	2, 3
Denmark	Danish Streamfauna Index	4
Finland	Finnish Multimetric Index	5, 6, 7
France	Global Biological Normalized Index or I_2M_2	8
Hungary	Hungarian Multimetric Index	9
Lithuania	Lithuanian River Macroinvertebrate Index	10
Spain	Iberian Biological Monitoring Working Party; MBfindex for the Basque region	11, 12, 13
Sweden	Average Score Per Taxon and the DJ index	5, 14
UK	Whalley Hawkes Paisley Trigg (WHPT) Average Score Per Taxon	15

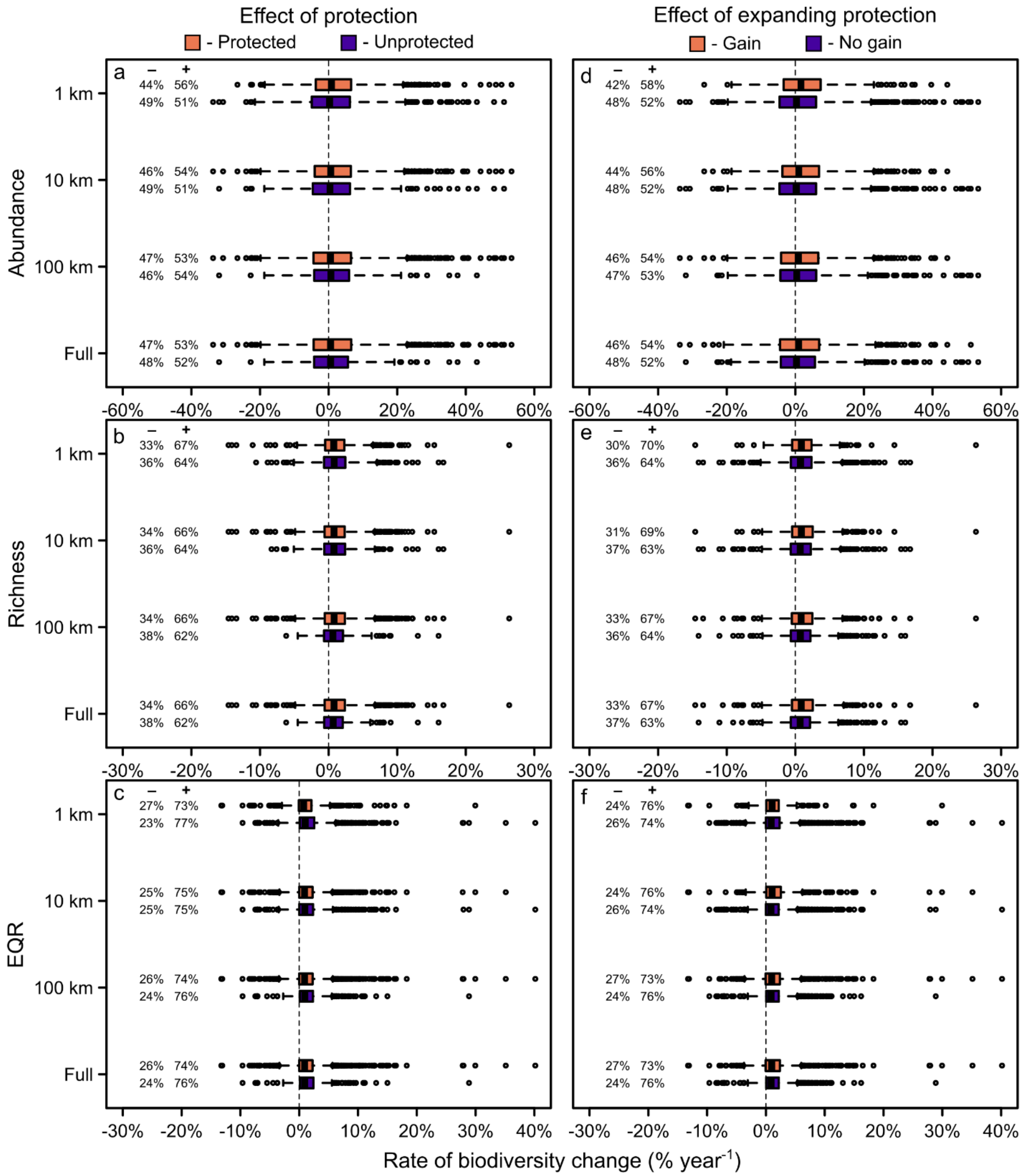

Supplementary Fig. 1 Rate of temporal change (% year^{-1}) in (a, d) abundance, (b, e) richness, and (c, f) EQRs in (a–c) protected versus unprotected sites, and (d–f) in sites that gained versus did not gain upstream protected area cover (protected/gain = orange; unprotected/no gain = purple), for the 1 km, 10 km, 100 km, and full upstream areas. Left and right edges of boxes represent the first and third quartiles, respectively, with a dark, horizontal line as the median. Whiskers encompass data points that lie within 1.5 times the inner quartile range, data outside this range are shown as separate points. Percentages to the left of the boxes indicate the

proportion of sites in each group that exhibit declining (-) or increasing (+) biodiversity trends. Note the changing scales of the x-axes, and that the sample sizes in the panels are the same as shown in Fig. 2.

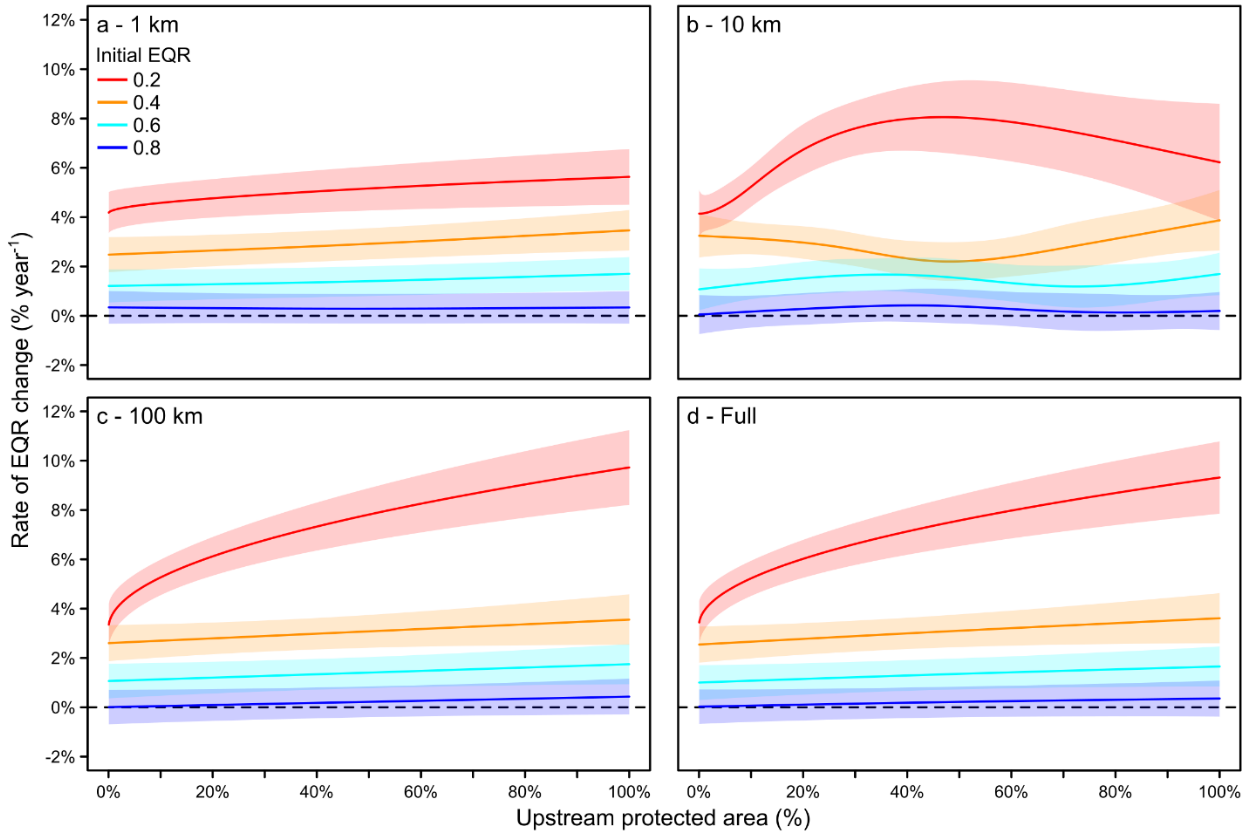

Supplementary Fig. 2 Influence of increasing the amount of upstream protected area cover (%) on the rate of temporal change (% year^{-1}) in ecological quality (as the Ecological Quality Ratio; EQR) for the (a) 1 km, (b) 10 km, (c) 100 km, and (d) full upstream areas. Lines show the best-fit relationships, with shaded areas as the 95% confidence intervals, based on the respective generalized additive mixed models. Line and shading color illustrate how relationships depend on initial ecological quality (as the initial EQR) using example initial EQRs of 0.2 (red), 0.4 (orange), 0.6 (light blue), and 0.8 (dark blue). Lower EQRs indicate initially poorer quality communities characteristic of rivers experiencing higher human impacts.

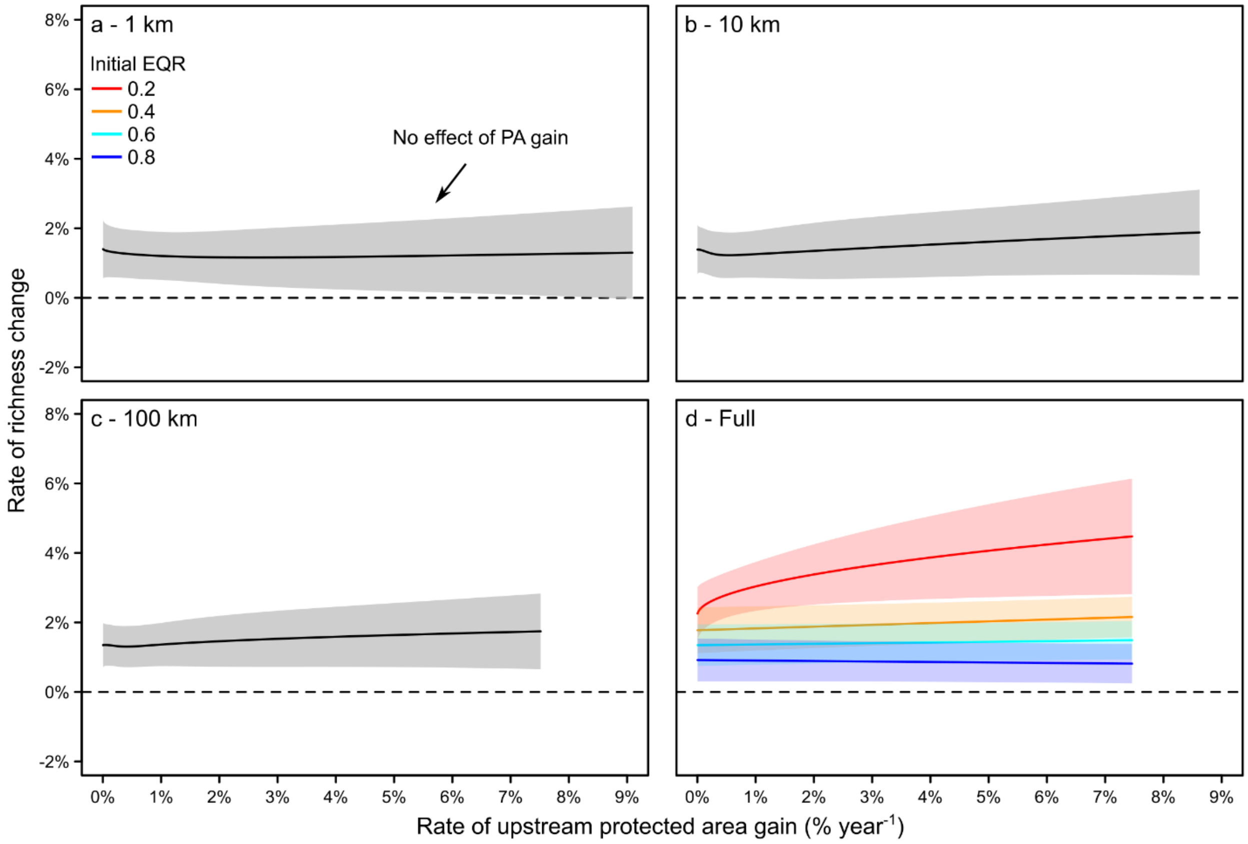

Supplementary Fig. 3 Influence of increasing the rate of gain of upstream protected area cover (% year^{-1}) on the rate of temporal change in richness (% year^{-1}) for the (a) 1 km, (b) 10 km, (c) 100 km, and (d) full upstream areas. Lines show the best-fit relationships, with shaded areas as the 95% confidence intervals, based on the respective generalized additive mixed models. Line and shading color illustrate relationships that depend on initial ecological quality (as the initial Ecological Quality Ratio; EQR) using example initial EQRs of 0.2 (red), 0.4 (orange), 0.6 (light blue), and 0.8 (dark blue). Lower EQRs indicate initially poorer quality communities characteristic of rivers experiencing higher human impacts. Black lines and grey shading indicate non-significant ($P < 0.05$) relationships. Note best-fit relationships are only shown up to the maximum rate of protected area gain observed at each upstream area.

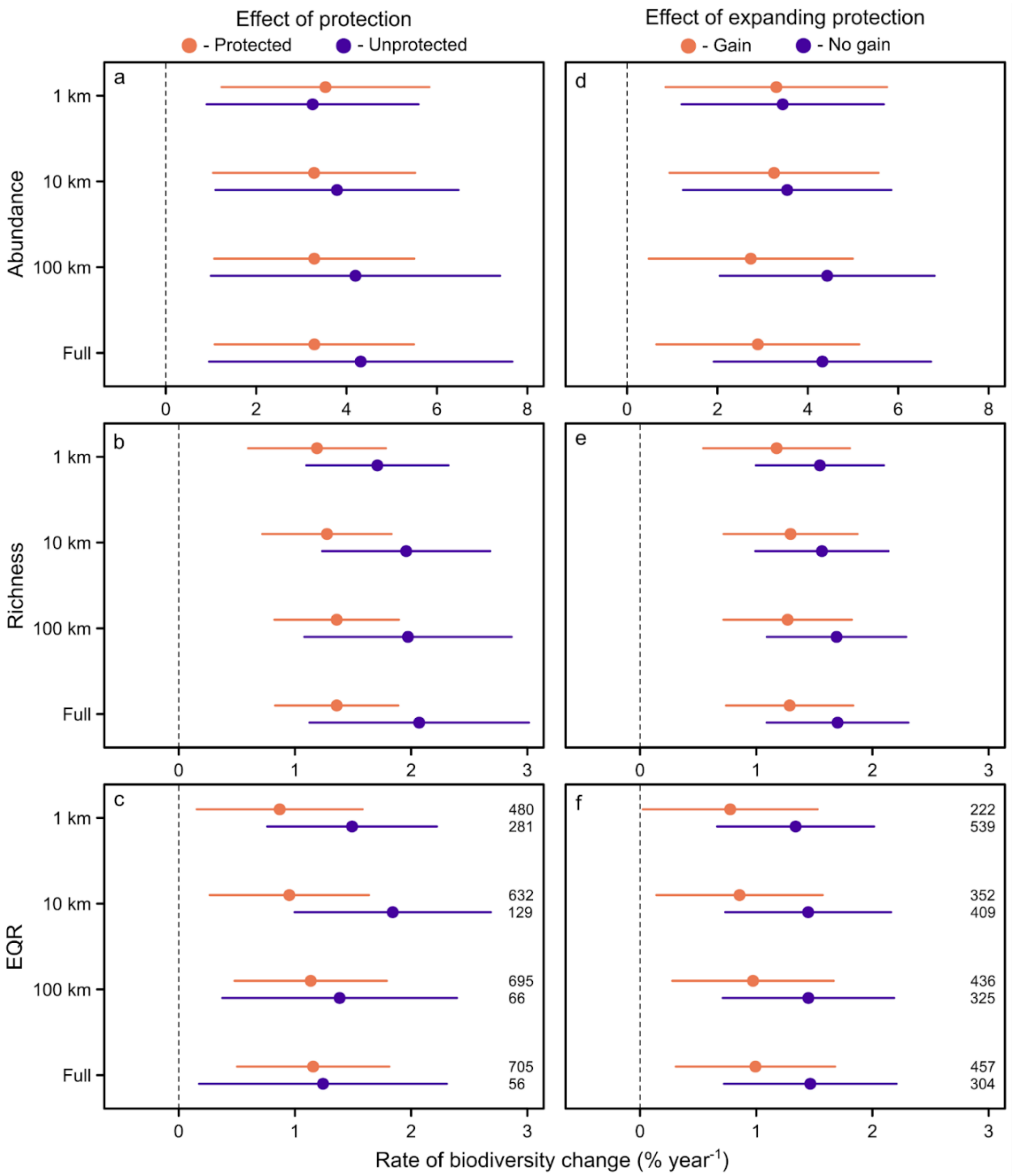

Supplementary Fig. 4 Rate of temporal change in (a, d) abundance, (b, e) richness, and (c, f) ecological quality (as the Ecological Quality Ratio; EQR) in (a–c) protected and unprotected sites, and (d–f) in sites that gained and did not gain upstream protected area cover, for the 1-km, 10-km, 100-km, and full upstream scales. Included sites were restricted to only those which provided a mixture of family, genus, and species-level information (761 sites total). Points show the predicted group mean based on the respective linear mixed model, with lines as 95% confidence intervals. Asterisks indicate significant ($P < 0.05$) differences between groups. Numbers in (c, f) indicate the number of sites out of 761 total in each group, and these same sample sizes apply to (a, b) and (d, e).

Supplementary note 1 – Protected area categories

The International Union for Conservation of Nature (IUCN) recognizes six official PA categories, labeled from I–VI, which range from those that exert stricter limits on human activity (e.g., categories I/II) to those that allow for multiple human uses, such as land development and resource extraction (e.g., categories V/VI). The Protected Planet database also includes PAs without an official IUCN designation, which can include strictly and less strictly protected areas. We initially considered examining differences among these different PA groups, specifically 'strict' (I–II), 'multi-use' (III–VI), and 'other' (those without a designation; following similar categorizations from ref. 16), given that stricter PAs may experience lower human impacts. Unfortunately, our European dataset includes comparatively few sites with strict PAs. For example, strict PAs alone only occur in 23 of the total 1,472 sites with upstream PAs. Similarly, few sites gain strict PA cover, with most gains driven by multi-use/other PAs (see Supplementary Fig. 5). This problem of the generally low occurrence of strict PAs applies not only to our dataset, but to many individual European countries (e.g., the UK[17]) and globally as well[18].

Our dataset still allowed for a comparison between biodiversity responses in protected sites with *any* strict upstream PA cover versus protected sites without, and we found no consistent effect of protection strictness (see Supplementary Fig. 6). However, we elected not to include this analysis in the main text for two reasons. First, sites with any upstream strict PAs generally also have upstream multi-use/other PAs, so this analysis captured the effect (or lack thereof) of having a strict PA mixed in with other PA types rather than the effect of strict protection itself. Second, the low number of sites with any upstream strict PAs (see site numbers in Supplementary Fig. 6e, f) meant that many comparisons were not robust. Instead, we opted to discuss literature showing equivocal evidence for the effectiveness of stricter protection. This equivocacy is echoed by our Supplementary Fig. 6 results, but we caution against using these results as robust scientific evidence of such.

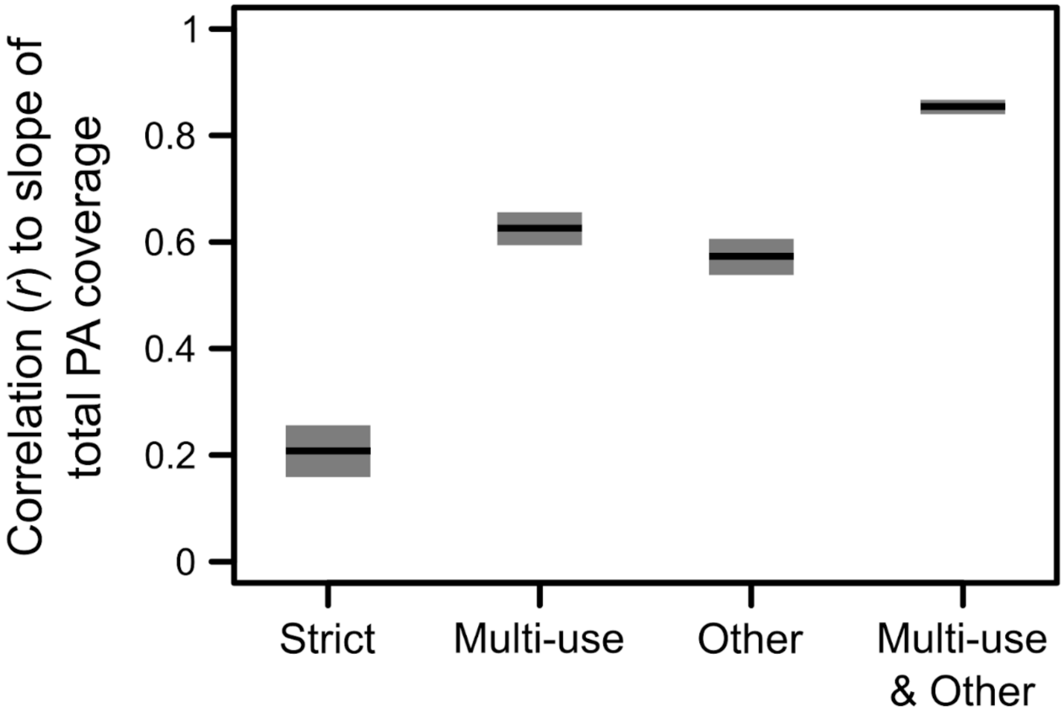

Supplementary Fig. 5 Pearson correlation (*r*) between temporal gains in total protected area (PA) cover and gains specifically in 'strict' (IUCN categories I–II), 'multi-use' (III–VI), and 'other' PA types (i.e., those without an official IUCN category). Correlations are shown as the mean (black line) and range (grey box) across the four upstream scales. These results indicate that gains in total PA cover are primarily driven by gains in multi-use and other PA types.

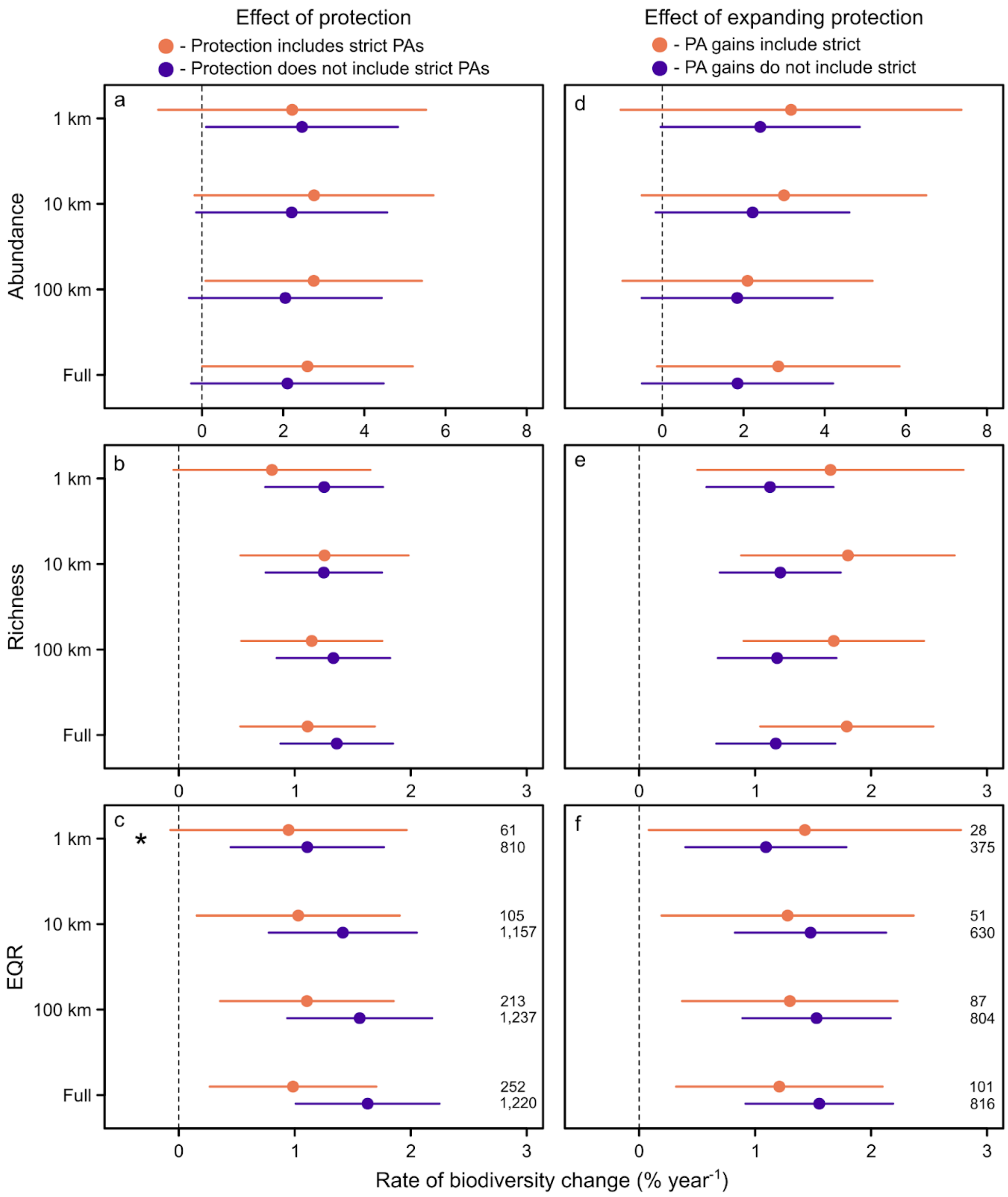

Supplementary Fig. 6 Rate of temporal change in (a, d) abundance, (b, e) richness, and (c, f) ecological quality (as the Ecological Quality Ratio; EQR) in (a–c) protected sites with and without strict protected areas, and (d–f) in sites that gained protected area cover where these gains included and did not include gains in strict protected area, for the 1-km, 10-km, 100-km, and full upstream scales. Points show the predicted rate based on the respective linear mixed model, with lines as 95% confidence intervals. Asterisks indicate significant ($P < 0.05$) differences between groups. Numbers in (c) and (f) indicate the number of sites in each group, which is the same for all metrics.

Supplementary references

1. Gabriels, W., Lock, K., De Pauw, N. & Goethals, P. L. M. (2010). Multimetric Macroinvertebrate Index Flanders (MMIF) for biological assessment of rivers and lakes in Flanders (Belgium). *Limnologica - Ecology and Management of Inland Waters*, *40*, 199–207.
2. Mičaník, T., Vyskoč, P., Prchalová, H., Polášek, M., Němejcová, D., Durčák, M., *et al.* (2020). Hodnocení stavu útvarů povrchových vod v České republice pro 3. plánovací období plánů povodí. *Vodohospodářské technicko-ekonomické informace*, *62*, 4–18.
3. Opatřilová, L., Němejcová, D., Zahrádková, S. & Kokeš, J. (2015). Assessment method for ecological status of rivers based on biological quality element benthic invertebrates in the Czech Republic. Presented at the XXII. Ogólnopolskie Warsztaty Bentologiczne, Karkonosze, Przesieka, p. 3.
4. Skriver, J., N. Friberg, and J. Kirkegaard. 2000. Biological assessment of running waters in Denmark: introduction of the Danish Stream Fauna Index (DSFI). *SIL Proceedings, 1922-2010*, *27*, 1822–1830.
5. Andersen, J. H., Aroviita, J., Carstensen, J., Friberg, N., Johnson, R. K., Kauppila, P., *et al.* (2016). Approaches for integrated assessment of ecological and eutrophication status of surface waters in Nordic Countries. *Ambio*, *45*, 681–691.
6. Aroviita, J., Koskenniemi, E., Kotanen, J. & Hämäläinen, H. (2008). A priori typology-based prediction of benthic macroinvertebrate fauna for ecological classification of rivers. *Environmental Management*, *42*, 894–906.
7. Aroviita, J., Mitikka, S. & Vienonen, S. (2019). Status classification and assessment criteria of surface waters in the third river basin management cycle. *Suomen ympäristökeskuksen raportteja*, No. 37/2019.
8. Mondy, C. P., Villeneuve, B., Archaimbault, V. & Usseglio-Polatera, P. (2012). A new macroinvertebrate-based multimetric index (I2M2) to evaluate ecological quality of French wadeable streams fulfilling the WFD demands: A taxonomical and trait approach. *Ecological Indicators*, *18*, 452–467.
9. Bozóki, T., Krasznai-Kun, E. Á., Csercsa, A., Várbíró, G. & Boda, P. (2018). Temporal and spatial dynamics in aquatic macroinvertebrate communities along a small urban stream. *Environmental Earth Sciences*, *77*, 559.
10. Šidagytė-Copilas, E. & Arbačiauskas, K. (2022). A multimetric macroinvertebrate index for the assessment of the ecological status of Lithuanian rivers. *Limnologica*, *97*, 126010.
11. Alba-Tercedor, J., Jáimez-Cuéllar, P., Álvarez, M., Avilés, J., Bonada, N., Casas, J., Mellado, A., Ortega, M., Pardo, I., Prat, N., Rieradevall, M., Robles, S., Sáinz-Cantero, C. E., Sánchez-Ortega, A., Suárez, Ma L., Toro, M., Vidal-Abarca, Ma R., Vivas, S., Zamora-Muñoz, C. (2002). Caracterización del estado ecológico de ríos mediterráneos ibéricos mediante el índice IBMWP (antes BMWP'). *Limnetica*, *21*, 175–185.
12. Munné, A. & Prat, N. (2009). Use of macroinvertebrate-based multimetric indices for water quality evaluation in Spanish Mediterranean rivers: an intercalibration approach with the IBMWP index. *Hydrobiologia*, *628*, 203–225.
13. URA (Agencia Vasca del Agua). (2014). *Protocolo de muestreo, análisis y evaluación de fauna bentónica macroinvertebrada en ríos vadeables*. Agencia Vasca del Agua.
14. Dahl, J. & Johnson, R.K. (2004). A multimetric macroinvertebrate index for detecting organic pollution of streams in southern Sweden. *Archiv für Hydrobiologie*, *160*, 487–513.
15. Paisley, M. F., Trigg, D. J. & Walley, W. J. (2014). Revision of the Biological Monitoring

Working Party (BMWP) score system: derivation of present-only and abundance-related scores from field data. *River Research and Applications*, *30*, 887–904.
16. Elleason, M., Guan, Z., Deng, Y., Jiang, A., Goodale, E. & Mammides, C. (2021). Strictly protected areas are not necessarily more effective than areas in which multiple human uses are permitted. *Ambio*, 50, 1058–1073.
17. Starnes, T., Beresford, A.E., Buchanan, G.M., Lewis, M., Hughes, A. & Gregory, R.D. (2021). The extent and effectiveness of protected areas in the UK. *Global Ecology and Conservation*, 30, e01745.
18. Guan, Z., Elleason, M., Goodale, E. & Mammides, C. (2021). Global patterns and potential drivers of human settlements within protected areas. *Environmental Research Letters*, 16, 064085.
