# Supplementary material for: Current protected areas provide limited benefits for European river biodiversity
Source: Nat Commun. 2025 Dec 17;16:11146. doi: 10.1038/s41467-025-67125-5 (PMC12711900; doi:10.1038/s41467-025-67125-5)
Supplement: Supplementary file 2 — Reporting Summary [file 41467_2025_67125_MOESM2_ESM.pdf]

nature portfolio

Corresponding author(s): James S. Sinclair

Last updated by author(s): Oct 29, 2025

# Reporting Summary

Nature Portfolio wishes to improve the reproducibility of the work that we publish. This form provides structure for consistency and transparency in reporting. For further information on Nature Portfolio policies, see our Editorial Policies and the Editorial Policy Checklist.

## Statistics

For all statistical analyses, confirm that the following items are present in the figure legend, table legend, main text, or Methods section.

| n/a | Confirmed | |
|---|---|---|
| ☐ | ☒ | The exact sample size (*n*) for each experimental group/condition, given as a discrete number and unit of measurement |
| ☐ | ☒ | A statement on whether measurements were taken from distinct samples or whether the same sample was measured repeatedly |
| ☐ | ☒ | The statistical test(s) used AND whether they are one- or two-sided<br>*Only common tests should be described solely by name; describe more complex techniques in the Methods section.* |
| ☐ | ☒ | A description of all covariates tested |
| ☐ | ☒ | A description of any assumptions or corrections, such as tests of normality and adjustment for multiple comparisons |
| ☐ | ☒ | A full description of the statistical parameters including central tendency (e.g. means) or other basic estimates (e.g. regression coefficient) AND variation (e.g. standard deviation) or associated estimates of uncertainty (e.g. confidence intervals) |
| ☐ | ☒ | For null hypothesis testing, the test statistic (e.g. *F*, *t*, *r*) with confidence intervals, effect sizes, degrees of freedom and *P* value noted<br>*Give P values as exact values whenever suitable.* |
| ☒ | ☐ | For Bayesian analysis, information on the choice of priors and Markov chain Monte Carlo settings |
| ☒ | ☐ | For hierarchical and complex designs, identification of the appropriate level for tests and full reporting of outcomes |
| ☒ | ☐ | Estimates of effect sizes (e.g. Cohen's *d*, Pearson's *r*), indicating how they were calculated |

*Our web collection on statistics for biologists contains articles on many of the points above.*

## Software and code

Policy information about availability of computer code

| | |
|---|---|
| Data collection | All data was collated for analyses using R version 4.3.2. |
| Data analysis | All data analyses were performed using R version 4.3.2. All code needed to repeat our analyses is publicly available from https://doi.org/10.6084/m9.figshare.25245430. |

For manuscripts utilizing custom algorithms or software that are central to the research but not yet described in published literature, software must be made available to editors and reviewers. We strongly encourage code deposition in a community repository (e.g. GitHub). See the Nature Portfolio guidelines for submitting code & software for further information.

## Data

Policy information about availability of data

All manuscripts must include a data availability statement. This statement should provide the following information, where applicable:

- Accession codes, unique identifiers, or web links for publicly available datasets
- A description of any restrictions on data availability
- For clinical datasets or third party data, please ensure that the statement adheres to our policy

All data needed to repeat our analyses are publicly available from https://doi.org/10.6084/m9.figshare.25245430.

## Research involving human participants, their data, or biological material

Policy information about studies with human participants or human data. See also policy information about sex, gender (identity/presentation), and sexual orientation and race, ethnicity and racism.

| | |
|---|---|
| Reporting on sex and gender | NA |
| Reporting on race, ethnicity, or other socially relevant groupings | NA |
| Population characteristics | NA |
| Recruitment | NA |
| Ethics oversight | NA |

Note that full information on the approval of the study protocol must also be provided in the manuscript.

# Field-specific reporting

Please select the one below that is the best fit for your research. If you are not sure, read the appropriate sections before making your selection.

☐ Life sciences ☐ Behavioural & social sciences ☒ Ecological, evolutionary & environmental sciences

For a reference copy of the document with all sections, see nature.com/documents/nr-reporting-summary-flat.pdf

# Ecological, evolutionary & environmental sciences study design

All studies must disclose on these points even when the disclosure is negative.

| | |
|---|---|
| Study description | We collated annual time series data on riverine invertebrate community composition at 1,754 sites across 10 European countries. |
| Research sample | The time series each consisted of at least seven individual years of data spanning a total duration of at least ten years, with sampling conducted at the same sites, during the same seasons (any three consecutive months), and using the same methods within sites through time. |
| Sampling strategy | Sites were selected based on which invertebrate communities were being monitored in their respective countries. The data collected from these sites also needed to satisfy our data requirements listed in the "Research sample" section above. |
| Data collection | Invertebrates were generally collected via multi-habitat kick-net samples collected from the river bottom. |
| Timing and spatial scale | Sites encompassed 10 European countries spanning across northern, central, and southern Europe. The time series across sites ranged between 1986 and 2022. Many sites provide data collected almost annually, although it is still common for time series to exhibit gaps of several years between samples. |
| Data exclusions | We excluded any time series that did not meet our "Research sample" requirements, and that did not provide the necessary information to quantify community abundance, richness, and ecological quality. |
| Reproducibility | No experiments were performed as part of this study. |
| Randomization | We controlled for both spatial/methodological relationships among sites and temporal relationships within sites. We controlled for spatial similarities by grouping data by provider, which typically grouped sites from the same country but some countries have multiple data providers. We controlled for temporal relationships by controlling for temporal autocorrelation among successive samples collected from the same site when calculating biodiversity change. |
| Blinding | Blinding was not relevant to this study because data acquisition required open contact among all of the ecologists and environmental managers that provided data. |

Did the study involve field work? ☐ Yes ☒ No

# Reporting for specific materials, systems and methods

We require information from authors about some types of materials, experimental systems and methods used in many studies. Here, indicate whether each material, system or method listed is relevant to your study. If you are not sure if a list item applies to your research, read the appropriate section before selecting a response.

## Materials & experimental systems

| n/a | Involved in the study |
| --- | --- |
| ☒ | ☐ Antibodies |
| ☒ | ☐ Eukaryotic cell lines |
| ☒ | ☐ Palaeontology and archaeology |
| ☐ | ☒ Animals and other organisms |
| ☒ | ☐ Clinical data |
| ☒ | ☐ Dual use research of concern |
| ☒ | ☐ Plants |

## Methods

| n/a | Involved in the study |
| --- | --- |
| ☒ | ☐ ChIP-seq |
| ☒ | ☐ Flow cytometry |
| ☒ | ☐ MRI-based neuroimaging |

# Animals and other research organisms

Policy information about studies involving animals; ARRIVE guidelines recommended for reporting animal research, and Sex and Gender in Research

| | |
| --- | --- |
| Laboratory animals | NA |
| Wild animals | No animals were collected for this specific study. Only data that had already been collected for other purposes in each region was used. |
| Reporting on sex | NA |
| Field-collected samples | No samples were collected or stored specifically for this study. |
| Ethics oversight | No ethical approval or guidance was required because no animals were collected for this study. |

Note that full information on the approval of the study protocol must also be provided in the manuscript.

# Plants

| | |
| --- | --- |
| Seed stocks | NA |
| Novel plant genotypes | NA |
| Authentication | NA |
